# The measurement of autoantibodies to insulin informs diagnosis of diabetes in a childhood population negative for other autoantibodies

**DOI:** 10.1111/dme.14979

**Published:** 2022-10-30

**Authors:** Claire L. Williams, Rachel J. Aitken, Isabel V. Wilson, Georgina L. M. Mortimer, Anna E. Long, Alistair J. K. Williams, The BOX Study Group, Kathleen M. Gillespie

**Affiliations:** ^1^ Diabetes and Metabolism, Bristol Medical School, University of Bristol, Southmead Hospital Bristol UK

**Keywords:** autoimmunity, islet autoantibodies, type 1 diabetes

## Abstract

**Aims:**

Some childhood type 1 diabetes cases are islet autoantibody negative at diagnosis. Potential explanations include misdiagnosis of genetic forms of diabetes or insufficient islet autoantibody testing. Many NHS laboratories offer combinations of three autoantibody markers. We sought to determine the benefit of testing for additional islet autoantibodies, including insulin (IAA) and tetraspanin 7 (TSPAN7A).

**Methods:**

Radiobinding assays (RBAs) were used to test for four islet autoantibodies in children with newly diagnosed type 1 diabetes (*n* = 486; 54.1% male; median age 10.4 years [range 0.7–18.0]; median duration 1 day [range −183 to 14]). Islet autoantibody negative children were tested for TSPAN7A using a luminescence‐based test. Where available, islet cell antibody (ICA) and human leucocyte antigen (HLA) data were considered.

**Results:**

Using three autoantibody markers, 21/486 (4.3%) children were autoantibody negative. Testing for IAA classified a further 9/21 (42.9%) children as autoantibody positive. Of the remaining 12 (2.5%) autoantibody negative children, all were TPAN7A negative, seven were ICA negative and one was positive for the protective variant DQB1*0602. One was subsequently diagnosed with Maturity Onset of Diabetes in the Young, but follow‐up was not available in all cases.

**Conclusions:**

Using highly sensitive assays, testing for three autoantibodies fails to detect islet autoimmunity in approximately 1/20 children diagnosed with type 1 diabetes. Testing for IAA in children <5 years and GADA in those >10 years was the most effective strategy for detecting islet autoimmunity. The ability to test for all islet autoantibodies should inform clinical decisions and make screening for monogenic diabetes more cost‐effective.

## Graphical abstract

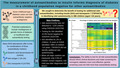

**What is already known?**

- All four major islet autoantibodies (GADA/IA‐2A/IAA/ZnT8A) are prevalent at diagnosis of type 1 diabetes in children. Only selected combinations are measured to identify cases for genetic screening.

**What has this study has found?**

- Testing for GADA/IA‐2A/ZnT8A using highly sensitive assays fails to detect islet autoimmunity in approximately 1/20 children diagnosed with type 1 diabetes.
- Testing for IAA identified autoimmunity in 9/21 (42.9%) children found negative by GADA/IA‐2A/ZnT8A testing, but 2.5% remain negative for all four islet autoantibodies as well as TSPAN7A.

**What are the implications of the study?**

- Robust testing for IAA in children will help inform clinical decision making in young children and make screening for monogenic diabetes more efficient.

## 1 INTRODUCTION

Autoantibodies to endogenous insulin (IAA), glutamate decarboxylase 65 (GADA), islet antigen‐2 (IA‐2A) and zinc transporter 8 (ZnT8A) are the primary biomarkers for predicting future autoimmune diabetes and are a distinctive clinical feature to aid classification at diagnosis. In individuals with newly diagnosed type 1 diabetes, at least 70% are positive for one or more of these islet autoantibodies^1^; prevalence of specific markers can vary by age, sex, genetic factors and diabetes duration at the time of detection.^2^

Prior to the development of antigen‐specific islet autoantibody tests (IAA, GADA, IA‐2A and ZnT8A), assays to detect islet cell cytoplasmic autoantibodies (ICA) by indirect immunofluorescence on fresh frozen human pancreas were developed.^3^ Positivity for ICA is not specific for antigen(s) but was shown to be highly prevalent in children with new‐onset type 1 diabetes.^4^ Current National Institute for Health and Care Excellence (NICE) guidelines do not recommend routine testing of autoantibodies in children, but testing in adults is endorsed to aid the classification of diabetes type.^5^, ^6^, ^7^ However, islet autoantibody screening at diagnosis in children is important to identify potential cases of monogenic diabetes (estimated to be approximately 2.5% of UK childhood cases)^8^ for subsequent genetic screening and optimal non‐insulin treatment strategies.

The performance and combination of islet autoantibody tests selected for analysis may contribute to incorrect identification of children as islet autoantibody negative.^9^, ^10^, ^11^, ^12^ The most widely performed method in type 1 diabetes research studies are radiobinding assays (RBAs) but due to use of radionuclides, safer and less labour‐intensive methods have been adopted in clinical practice. Currently, most NHS laboratories test for three autoantibody markers (GADA, IA‐2A and ZnT8A) through commercially available methods, commonly by enzyme‐linked immunosorbent assays (ELISAs). Testing of ICA is still used in some NHS laboratories and is often performed on commercially available primate or rodent pancreas. However, ICA assays are associated with poor specificity even when human pancreas is used and despite international standardisation efforts, low and variable inter‐laboratory concordance can occur.^7^, ^13^ For these reasons, the number of research laboratories conducting ICA assays has diminished markedly. The precise utilisation of ICA assays in NHS laboratories at present remains unknown.

Compared to other islet autoantibody markers, accurate testing of IAA at clinical onset can be difficult. First, IAA must be detected within 2 weeks of diagnosis prior to the initiation of insulin treatment as IAA cannot be differentiated from antibodies to injected insulin.^14^ Second, precise measurement of IAA is technically complex as levels are often low requiring very sensitive tests and, competitive displacement with recombinant human insulin is necessary to confirm IAA specificity. Third, there is currently no commercially available test that outperforms the IAA RBA. For instance, IAA ELISAs detect greater insulin binding in non‐diabetes‐related samples and fewer type 1 diabetes‐associated IAA compared to RBA,^15^ and can have poor sensitivity.^16^ Despite its high performance, IAA RBAs cannot be easily implemented into clinical laboratories, but a specialist islet autoantibody testing facility could support clinical laboratories by providing additional targeted testing of samples with unclear results to aid clinical decision making.

In the context of studying the natural history of autoimmunity in participants from the Bart's Oxford (BOX) study,^17^ we sought to evaluate the benefit of high‐performance IAA testing following GADA, IA‐2A and ZnT8A analysis, in children using RBAs in a specialised research laboratory. In children negative for all four biochemical autoantibody markers, we also tested for recently described autoantibodies to Tetraspanin‐7 (TSPAN7A).^18^

## 2 METHODS

### 2.1 Cohort description

**FIGURE 1:**
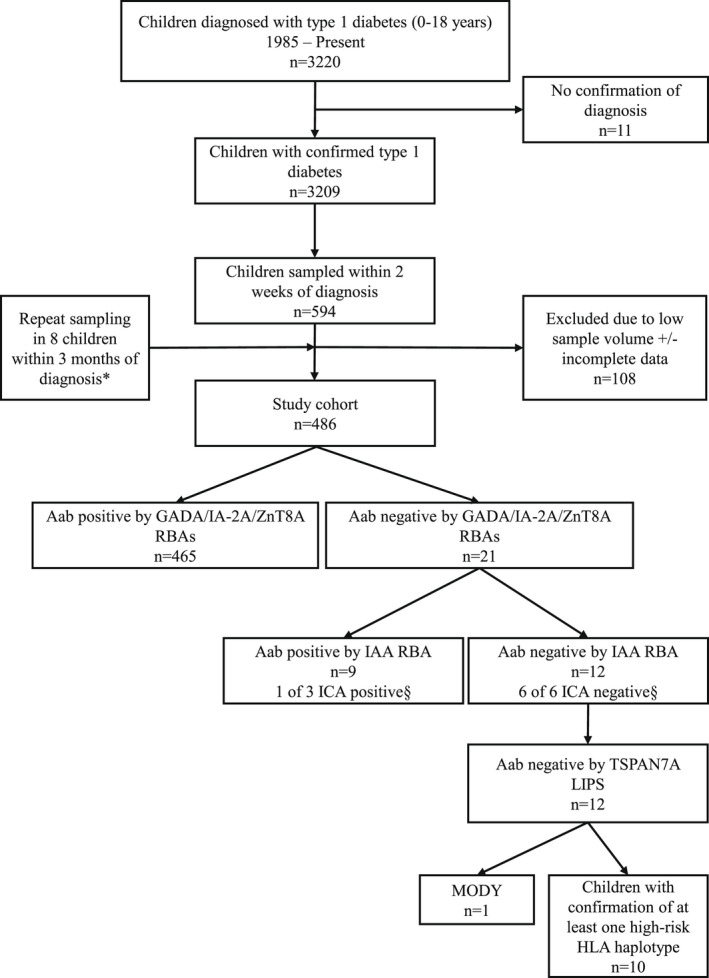
Study flow diagram. *In eight children, due to low sample volume, IAA was measured in samples obtained within 2 weeks of diagnosis and GADA/IA‐2A/ZnT8A was measured by repeat sampling within 3 months of diagnosis (median duration 67.5 days [range 15–91]). ^§^Available ICA data from historically conducted assays were considered in select analysis.

The population‐based BOX family study, established in 1985, has recruited individuals under 21 years with newly diagnosed type 1 diabetes (based on the World Health Organisation's criteria and a clinical requirement for insulin treatment) and their first‐degree relatives for long‐term follow‐up within the UK's former Oxford Regional Health Authority. The BOX study is currently approved by the South Central—Oxford C. National Research Ethics Committee. Participants provided informed, written consent and the study was performed according to the principles of the Declaration of Helsinki. All serum samples of sufficient volume, taken before or within 2 weeks of diagnosis, from children (aged < 18 years) with a clinical diagnosis of diabetes were selected for islet autoantibody testing (*n* = 486; 263 males [54.1%]; median age‐at‐diagnosis 10.4 years [range 0.7–18.0]; median diabetes duration 1 day [range − 183–14]). Of the 486 children, historical ICA data were available on 211 (43.3%) and were considered in select analyses (Figure 1). Characteristics of the cohort with available data for variables investigated are detailed in Table 1.

**TABLE 1:** Cohort description and available data from children with newly diagnosed type 1 diabetes

Variable	Number (%)
Sex (*n* = 486)	
Male	263 (54.1)
Female	223 (45.9)
Age at onset (*n* = 486)	
0–5 years	91 (18.7)
5–10 years	133 (27.4)
10–15 years	209 (43.0)
15–18 years	53 (10.9)
ICA (*n* = 211)^b^	
≥20 JDF‐U	139 (65.9)
<20 JDF‐U	72 (34.1)
HLA class II risk (*n* = 435)^a^	
High	154 (35.4)
Medium	205 (47.1)
Low	6 (1.4)
Neutral	70 (16.1)

Abbreviations: HLA, human leucocyte antigen; JDF‐U, juvenile diabetes foundation units.

^a^ High (*DR3‐DQ2* and *DR4‐DQ8)*, medium (presence of either *DR3‐DQ2* or *DR4‐DQ8* with a non‐protective HLA haplotype), neutral (presence of either *DR3‐DQ2* or *DR4‐DQ8* with a protective haplotype or haplotypes not associated with type 1 diabetes) and low (presence of one or two protective haplotypes in the absence of *DR3‐DQ2* or *DR4‐DQ8*) HLA susceptibility/protective haplotypes.

^b^ ICA was historically tested on fresh frozen O‐negative human pancreas.

### 2.2 Islet autoantibody determination

Autoantibodies to GAD65, IA‐2, ZnT8 (arginine [R] and tryptophan [W] to detect major ZnT8A variants [ZnT8RA/ZnT8WA]),^19^ and endogenous insulin were detected by RBAs, described previously.^20^, ^21^, ^22^ Logarithmic standard curves were used to determine units of autoantibody level and healthy populations were used to set positivity thresholds for GADA, IA‐2A, ZnT8A and IAA. Autoantibodies to TSPAN7 were detected using a luminescence immunoprecipitation system (LIPS) assay as previously described.^18^ The sensitivity at 95% specificity of these assays was assessed in the 2020 Islet Autoantibody Standardisation Program: 78% GADA, 74% IA‐2A, 70% ZnT8RA, 56% ZnT8WA, 62% IAA and 56% TSPAN7A. The ICA assay was conducted as previously described,^3^ and where data were available, a positivity threshold of 20 Juvenile Diabetes Foundation units (JDF‐U) was used to improve disease specificity.^13^

### 2.3 Human leucocyte antigen (HLA) class II genetic analysis

All available DNA samples were extracted from whole blood or mouth swab samples and whole‐genome amplified (Illustra GenomiPhi V2 DNA amplification kit; GE Healthcare). HLA class II alleles were determined in 436 (89.7%) children using polymerase chain reaction (PCR) sequence‐specific primers (SSP) as described previously,^23^ and categorised into four risk groups based on the presence of susceptibility or protective haplotypes^24^; high (presence of both DRB1*03‐DQA1*05:01‐DQB1*02:01 [*DR3‐DQ2*] and DRB1*04‐DQA1*03:01‐DQB1*03:02 [DR4‐DQ8]), medium (presence of *DR3‐DQ2* or *DR4‐DQ8* with a non‐protective HLA haplotype), neutral (presence of *DR3‐DQ2* or *DR4‐DQ8* with a protective haplotype [DR15]‐DQB1*0602, [DR13]‐DQB1*0603, [DR5]‐DQA1*05‐DQB1*0301, [DR7]‐DQA1*0201‐DQB1*0303, or haplotypes not associated with susceptibility/protection) and low (presence of one or two protective haplotypes in the absence of *DR3‐DQ2* or *DR4‐DQ8*).

### 2.4 Statistical analysis

Data were analysed and graphed using GraphPad PRISM (v.9.1.0; GraphPad Software). Proportions were compared using Chi‐squared X^2^ or Fisher's exact tests where appropriate. Mann–Whitney U tests were used to compare median IAA levels between single and multiple IAA‐positive children. One‐way Kruskal–Wallis test was used to compare median islet autoantibody titres between categories of age. In all analyses, a two‐tailed *p*‐value <0.05 was considered significant.

## 3 RESULTS

### 3.1 GADA, IA‐2A and ZnT8A are not detected in approximately one in twenty of children diagnosed with type 1 diabetes

Testing of GADA, IA‐2A and ZnT8A identified 465 (95.7%) children positive for at least one autoantibody. Of 465 autoantibody positive children, 397 (85.4%) were multiple autoantibody positive (≥2 autoantibodies), with 238 (51.2%) positive for all three autoantibodies and 68 (14.6%) were positive for a single autoantibody, where GADA was most common (43 [9.2%] GADA; 16 [3.4%] IA‐2A; 9 [1.9%] ZnT8A). A total of 21 children (4.3%) remained autoantibody negative.

### 3.2 Testing for IAA provides an autoimmune classification of diabetes in 42.9% of children negative for all other islet autoantibodies and improves identification of multiple autoantibody positive children by 8%

**FIGURE 2:**
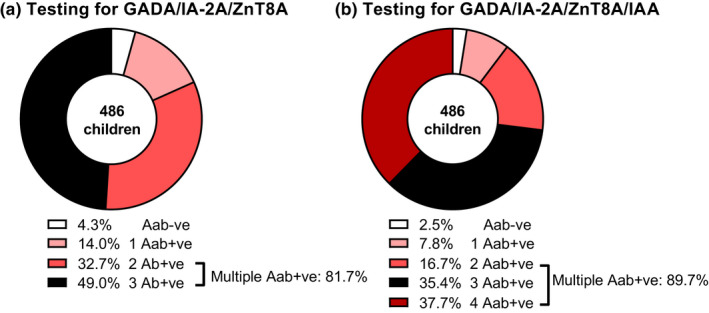
Comparison of GADA/IA‐2A/ZnT8A (a) and GADA/IA‐2A/ZnT8A/IAA (b) testing strategies. Testing for IAA provides an autoimmune classification of diabetes in 42.9% of children found autoantibody negative by GADA/IA‐2A/ZnT8A testing and identifies an additional 8.0% children positive for multiple (≥2) autoantibodies. The inclusion of IAA decreased the proportion of children found biochemical autoantibody from 4.3% to 2.5%.

Of 21 children found autoantibody negative by GADA/IA‐2A/ZnT8A testing, 9 (42.9%) were single IAA positive with 8 (88.9%) diagnosed <10 years, providing an autoimmune classification of diabetes. Of these 9 children, 3 had available ICA data which included one child who was ICA positive (80 JDF‐U; aged 4 years at diagnosis). Testing for IAA also increased the number of children found multiple autoantibody positive by 8.0% from 397 (81.7%) to 436 (89.7%), with 183/486 (37.7%) children positive for all four islet autoantibodies. In children positive for IAA, the median IAA level was not significantly different between single or multiple autoantibody IAA‐positive children (*p* > 0.05; Figure S1). The inclusion of IAA reduced the number of children found autoantibody negative from 21 (4.3%) to 12 (2.5%) in the total cohort of children (Figure 2a versus 2b). The islet autoantibody profiles in all 486 children are detailed in Table S1.

### 3.3 Testing for IAA is most likely to benefit the classification of autoimmune diabetes in young (<5 years) children

**FIGURE 3:**
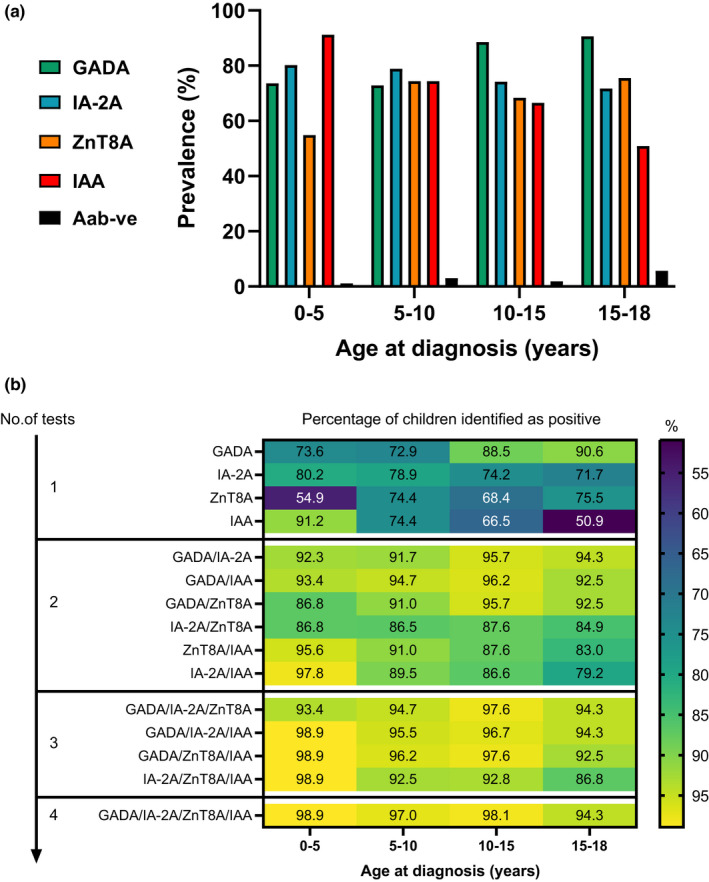
The prevalence and sensitivity of autoantibody markers by age at diagnosis and the number/combination of tests performed. (a) The prevalence of most islet autoantibodies are associated with age at diagnosis; GADA increased with age > 10 years (*p* = 0.00016), IAA decreased with age > 10 years (*p* = 6.59 × 10^−7^) and is highest in children aged <5 years, and ZnT8A was higher in children aged >5 years (*p* = 0.011). There was no relationship between age at diagnosis and prevalence of IA‐2A (*p* = 0.486) or absence of islet autoantibodies under or over 10 years (*p* = 0.99). (b) Overall, the sensitivity increases and is generally highest when all four islet autoantibodies are tested independent of age. However, as a single test, IAA identifies the highest percentage of children <5 years. While GADA as a single test, identifies the highest percentage of children diagnosed >10 years.

Islet autoantibody positivity is associated with age‐at‐diagnosis (Figure 3a); GADA increased with age > 10 years (*p* = 0.00016), IAA decreased with age > 10 years (*p* = 6.59 × 10^−7^) and ZnT8A increased >5 years (*p* = 0.011). There was no relationship between age at diagnosis and prevalence of IA‐2A (*p* = 0.486) or absence of islet autoantibodies (<10 years versus >10 years, *p* = 0.99).

The benefit of testing for IAA was greatest in children diagnosed <5 years. Of 6 children diagnosed <5 years negative for GADA/IA‐2A/ZnT8A, only one child (aged 1.9 years; 16.7%) remained islet autoantibody negative when IAA was also tested. In contrast, of 7 GADA/IA‐2A/ZnT8A negative children diagnosed 5–10 years and 8 diagnosed 10–18 years, 4 (57.1%) and 7 (87.5%), respectively, remained islet autoantibody negative. When IAA level was considered, the median level decreased as a function of age at diagnosis (*p* < 0.0001; Figure S2). In autoantibody‐positive children, levels of other autoantibodies were not associated with age at diagnosis (*p* > 0.05).

### 3.4 The optimal testing strategy of islet autoantibodies varies according to age at diagnosis in a childhood population; IAA <5 years and GADA >10 years

In general, identifying islet autoimmunity increases with the number of autoantibody markers measured independent of age, with ≥3 autoantibody tests achieving the highest sensitivity (range 97.0%–98.9%, Figure 3b). In the whole cohort of children, GADA achieved the greatest sensitivity, identifying 81.7% of children, followed by IA‐2A (76.3%), IAA (71.6%) and ZnT8A (68.3%), but age is an important consideration.

As a single test, IAA achieved the highest sensitivity in children aged <5 years, with 83/91 (91.2%) positive for IAA. In this age group, testing for IAA > IA‐2A ± ZnT8A > GADA appears logical with all four tests achieving 98.9% sensitivity. However, in children aged >10 years, IAA testing should be substituted for GADA. As a single test, GADA achieved the highest sensitivity in children >10 years, identifying >88%. Beyond this age, testing for GADA>IA‐2A/ZnT8A > IA‐2A + ZnT8A, is the optimal strategy with all markers achieving 94.3%–98.1% sensitivity. In children aged 5–10 years, no single marker was superior in detecting islet autoimmunity. Additionally, for participants aged 15–18 years, testing for GADA ± IA‐2A performed as well as testing for three or four islet autoantibodies.

### 3.5 Analysis of ICA, TSPAN7A and HLA class II risk did not help define autoimmune diabetes in the children who remained islet autoantibody negative

The 12 autoantibody negative children with diabetes (2.5% of cohort) were also negative when tested for TSPAN7A. ICA data were available from six children but only one child was borderline positive (19 JDF‐U). Type 1 diabetes HLA risk profiles from 10 children confirmed that three were high risk, three were medium risk and four were neutral risk. When available HLA data on 435 islet autoantibody positive and 10 islet autoantibody negative children were considered, there was no difference in the overall risk profiles, although one child was positive for the protective variant DR15‐DQB1*0602 (Figure S3a vs. S3b).

Availability of clinical follow‐up data from these children was limited, but one child was subsequently diagnosed with Maturity Onset of Diabetes in the Young (MODY). Of the remaining 11, three children had additional characteristics to support an autoimmune profile; one child positive for DR3‐DQ2/DR15‐DQB1*0602 was subsequently diagnosed with coeliac disease supporting an autoimmune pathogenesis despite the protective HLA haplotype; one child had undetectable post‐diagnosis C‐peptide, and one child had a first‐degree relative (father) with type 1 diabetes who remained IA‐2A positive 38 years after diagnosis. The remaining eight children (1.6% of the original cohort) represent good candidates for genetic screening for monogenic diabetes.

## 4 DISCUSSION

Using high‐performance islet autoantibody tests, we showed that after screening for GADA, IA‐2A and ZnT8A, approximately 1 in 20 children diagnosed with type 1 diabetes had no proven cause for their diabetes. If there is clinical suspicion of a monogenic form of diabetes, the absence of GADA, IA‐2A and ZnT8A would be sufficient justification to merit genetic screening. As IAA are often the first islet autoantibody marker to develop in early life,^25^ and are most prevalent in children with type 1 diabetes,^20^, ^26^ (when samples had been obtained less than 2 weeks from diagnosis), we tested for IAA using a high‐performance RBA to determine whether an autoimmune pathogenesis could be proven in additional children. IAA measurement increased multiple autoantibody positivity and provided an autoimmune classification of diabetes in an additional nine children. The remaining 12 children without a cause for their diabetes were tested for the most recently discovered autoantibody, TSPAN7A^18^; no additional cases of autoimmunity were identified. Subsequent analysis of ICA data from six participants was not helpful; only one child was borderline positive for ICA. Through a request from their consultant, one child underwent genetic screening and a MODY diagnosis was confirmed. One child was positive for the major protective variant DR15‐DQB1*0602 which occurs in only 1% of type 1 diabetes cases but this child developed coeliac disease as well as diabetes, supporting an autoimmune pathogenesis. Autoimmunity was also supported in one child where post‐diagnosis C‐peptide data were available and one child had a father with islet autoantibody positive type 1 diabetes. The highest risk HLA combination was observed in two children possibly suggesting these children are likely to have type 1 diabetes, but this combination is present in 3% of the general population making interpretation difficult. This study proves that with careful islet autoantibody testing strategies using high‐performance tests, islet autoimmunity was proven in 474 (97.5%) of cases and likely in an additional three cases leaving only nine cases (1.9%) for genetic screening, one of whom has been screened and found positive for MODY.

Characteristics for genetic screening have typically included onset before the age of 25–35 years, lack of insulin dependency (as shown by treatment or C‐peptide measurement), absence of obesity or other signs of insulin resistance, dominant inheritance over several generations and the absence of islet autoantibodies associated with type 1 diabetes.^27^ A recent report that involved autoantibody testing in almost 4000 children with a diagnosis of diabetes (1–18 years using American Diabetes Association criteria) from Sweden showed that the identification of islet autoantibody negative children was key to differentiating type 1 diabetes and referring children for genetic screening, which increased the efficiency of identifying MODY.^28^ Intriguingly 12% in this cohort tested negative for GADA, IA‐2A, ZnT8A and IAA compared with only 2.5% in our cohort. This may have been influenced by the differences in assays used and/or the Swedish study incorporating all children with a diagnosis of diabetes while the children in our study were diagnosed with type 1 diabetes.

Testing for IAA in our study approximately halved the number of children that would be referred for genetic screening but in the Swedish study, IAA measurement only reduced the number of patients with paediatric diabetes who were autoantibody negative from 13% to 12%. Although the mean age of participants was 10.1 years (SD 4.4) in the Swedish study and 10.4 years (range 0.7–18.0) in the UK study so similar overall, our demonstration of lower prevalence of IAA in children with an older age at diagnosis may account for some of this difference. A high frequency (25%) of IAA loss in children before diagnosis has been reported in birth cohort studies and this was dependent on the duration of the prediabetic period from IAA seroconversion, which is presumably longer for children diagnosed older.^26^

The UK‐based ADDRESS‐2 study also reported islet autoantibody data at diagnosis,^29^ but IAA could not be included because sampling was not within 2 weeks of diagnosis as required to avoid detection of antibodies to injected insulin. In 2011, Vermeulen and colleagues from the Belgian Diabetes Registry (BDR) reported results from 655 new‐onset cases (insulin treated, sampled <1 week from diagnosis and based on American Diabetes Association criteria) aged <40 years (median age 15 years [IQR 9–26]).^30^ The comparison between GADA/IA‐2A/IAA and GADA/IA‐2A/ZnT8A islet autoantibody testing panels indicated that IAA identified an additional 17 (10%) of children aged 0–9 years (*n* = 170), which is a lower frequency than we identified in BOX, but this is likely to reflect an older cohort at diagnosis and IAA assay differences. Supporting the data reported in this study, Vermeulen et al.'s data using the two testing panels also suggested that the sensitivity of IAA decreases with age as IAA did not identify additional islet autoimmunity in children aged 10–19 years (*n* = 223). However, we are unable to make direct comparisons between the children studied in BOX and the BDR due to the categorisation of age and islet autoantibody testing panels.

The optimal strategy to prove autoimmunity or identify possible genetic cases of diabetes is to test for all four islet autoantibodies. If cost‐saving strategies are required, our study shows that those making clinical decisions regarding genetic screening would be best incorporating IAA testing in young children (<5 years) at diagnosis of diabetes and children >10 years should be screened for GADA. This supports other reports that suggest autoantibody combinations present at diagnosis could be assembled into groups associated with either IAA or GADA.^26^ The increasing prevalence of GADA in older ages at diagnosis, particularly in adult‐onset autoimmune diabetes, is well‐known and was the most common marker overall in the study cohort. In children aged 15–18 years, testing for GADA ± IA‐2A performed as well as, testing for three or four islet autoantibodies. To our knowledge, this is first study to incorporate testing for TSPAN7A but only the samples negative for other islet autoantibodies were tested. In future, wider testing of TSPAN7A is required to fully define its usefulness above testing for GADA/IA‐2A/ZnT8A and IAA.

The clinical utility of islet autoantibody testing is likely to increase in the future. Our study shows that 4% of children are autoantibody negative when GADA/IA‐2A/ZnT8A are considered. In the Swedish study, 13% were autoantibody negative using the same panel.^28^ In the UK‐based ADDRESS‐2 cohort (*n* = 1778, aged ≥5 years within 6 months of diagnosis, 38% <17 years), 15% were GADA/IA‐2A/ZnT8A negative, with higher proportions at older ages, in males, and non‐white ethnicity.^29^ Our data support the observation that autoantibody negativity is more common in older age and while we identified a higher proportion of autoantibody positivity, we were unable to assess the effects of sex and ethnicity, due to small numbers and a predominantly white cohort. Additionally, we had limited clinical data from the children investigated; the ADDRESS‐2 study found no difference in presenting symptoms between autoantibody positive and negative participants across a wide age range, including adults.^29^ The Swedish study showed that modestly raised glucose levels also inform MODY screening strategies.^28^

Overall, we describe a pragmatic and cost‐effective approach to classifying diabetes in children using high‐performance islet autoantibody testing. We show that in children with a clinical diagnosis of type 1 diabetes, islet autoimmunity can be proven in >97.5% leaving a small proportion of children to be considered for genetic screening where costs are 10‐fold greater than islet autoantibody screening.

## AUTHORS' CONTRIBUTIONS

CLW, AEL and KGM contributed to the original idea and designed the study; CLW, AJKW, GLMM, RJA and IVW designed and performed data collection; CLW, GLMM, AEL and KMG performed data analysis and wrote the manuscript; all authors contributed to data interpretation, manuscript revision and approved the final manuscript. KMG is responsible for the integrity of the work as a whole.

## CONFLICTS OF INTEREST

The authors declare that there is no duality of interest associated with this manuscript.

## ETHICS STATEMENT

The Bart's Oxford study is currently approved by the South Central—Oxford C. National Research Ethics Committee. Participants provided informed, written consent and the study was performed according to the principles of the Declaration of Helsinki.

## Supporting information


Figure S1

Figure S2

Figure S3

Table S1

## Data Availability

Data supporting the conclusions reported in this manuscript are available upon reasonable request.
