# Supplementary figures and images for: The measurement of autoantibodies to insulin informs diagnosis of diabetes in a childhood population negative for other autoantibodies
Source: Diabet Med. 2022 Oct 30;39(12):e14979. doi: 10.1111/dme.14979 (PMC9827938; doi:10.1111/dme.14979)

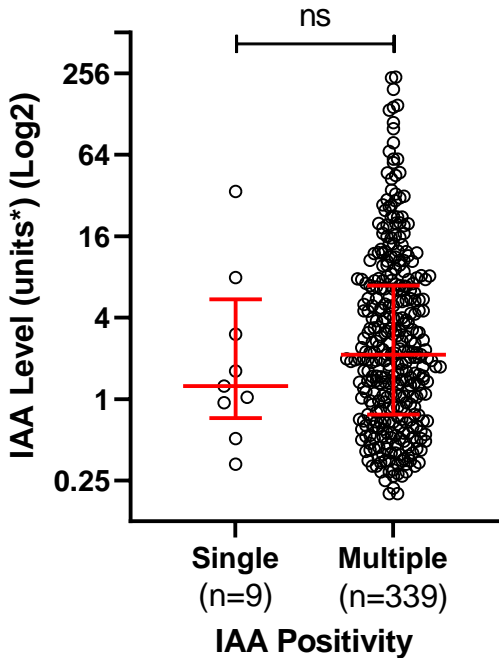

Supplement: Supplementary file 1 — Figure S1 [file DME-39-0-s004.pdf]

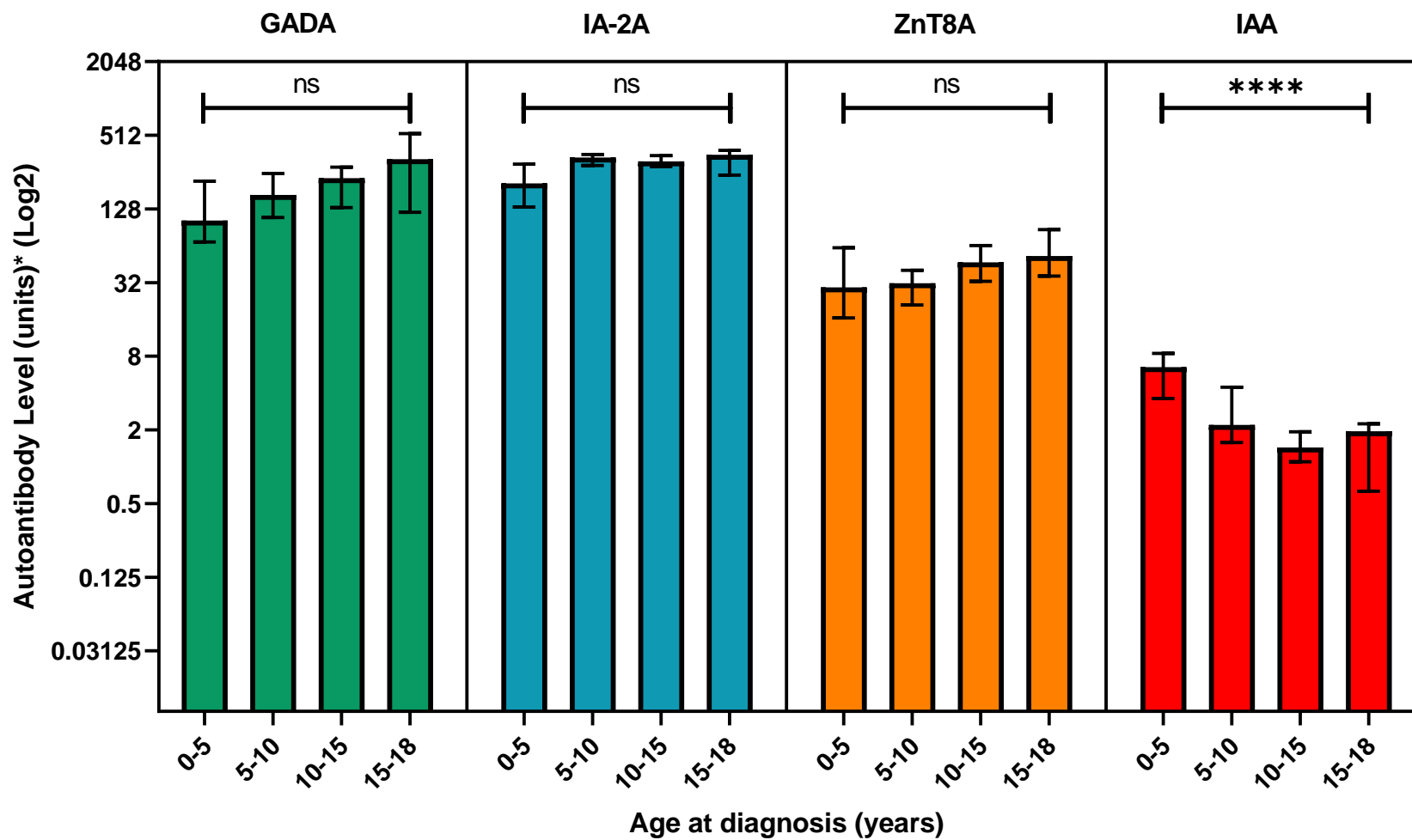

Supplement: Supplementary file 2 — Figure S2 [file DME-39-0-s003.pdf]
