# Supplementary material for: The measurement of autoantibodies to insulin informs diagnosis of diabetes in a childhood population negative for other autoantibodies
Source: Diabet Med. 2022 Oct 30;39(12):e14979. doi: 10.1111/dme.14979 (PMC9827938; doi:10.1111/dme.14979)
Supplement: Supplementary file 3 — Figure S3 [file DME-39-0-s002.pdf]

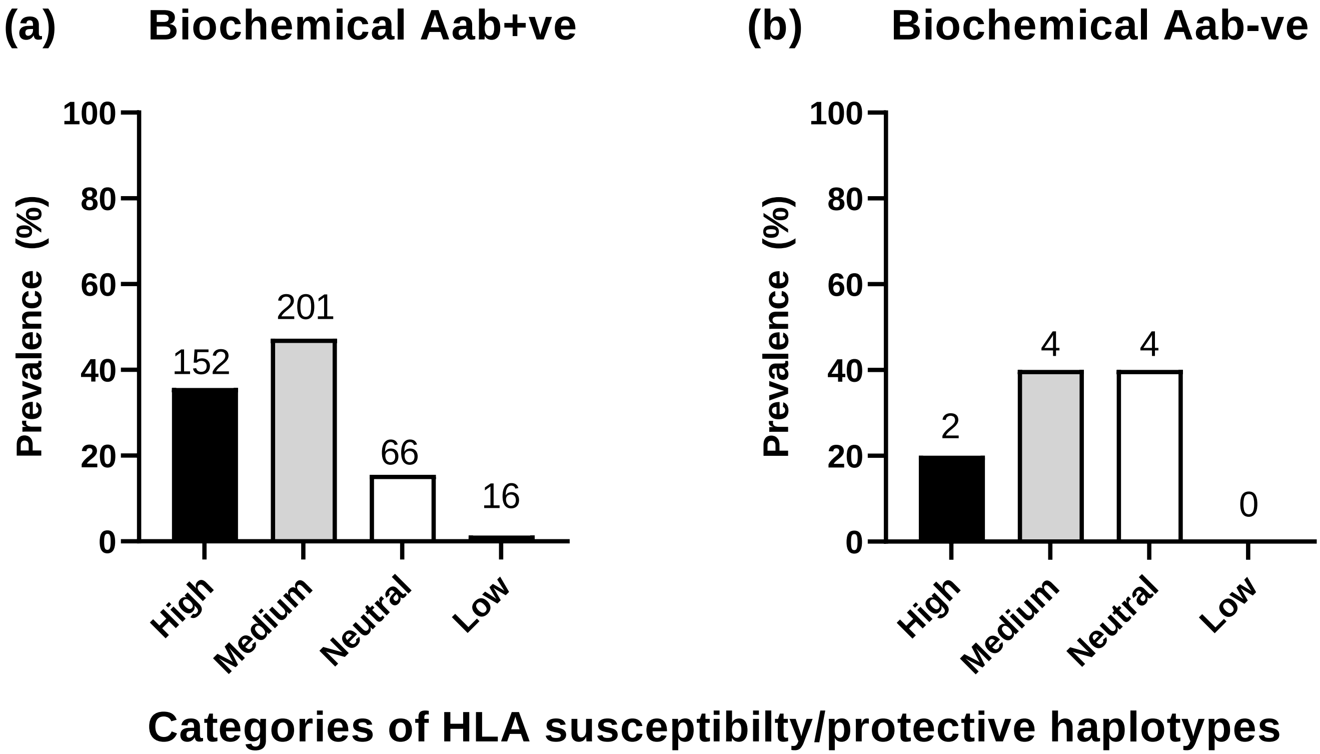
(a)
Biochemical Aab+ve
(b)
Biochemical Aab-ve
Prevalence (%)
100
80
60
40
20
0
152
201
66
16
2
4
4
0
High
Medium
Neutral
Low
Categories of HLA susceptibilty/protective haplotypes
