# Supplementary material for: The measurement of autoantibodies to insulin informs diagnosis of diabetes in a childhood population negative for other autoantibodies
Source: Diabet Med. 2022 Oct 30;39(12):e14979. doi: 10.1111/dme.14979 (PMC9827938; doi:10.1111/dme.14979)
Supplement: Supplementary file 4 — Table S1 [file DME-39-0-s001.pdf]

ESM Table 1 – Complete islet autoantibody profiles in all 486 children.

Islet Autoantibody Profile	Number	Frequency (%)
**One autoantibody**		
**GADA**	21	4.3
**IA-2A**	3	0.6
**ZnT8A**	5	1.0
**IAA**	9	1.9
**Total single autoantibody positive**	38	7.8
**Two autoantibodies**		
**GADA/IA-2A**	15	3.1
**GADA/ZnT8A**	17	3.5
**GADA/IAA**	22	4.5
**IA-2A/ZnT8A**	10	2.1
**IA-2A/IAA**	13	2.7
**ZnT8A/IAA**	4	0.8
**Three autoantibodies**		
**GADA/IA-2A/ZnT8A**	55	11.3
**IAA/IA-2A/ZnT8A**	33	6.8
**IAA/GADA/ZnT8A**	25	5.1
**IAA/GADA/IA-2A**	59	12.1
**Four autoantibodies**		
**GADA/IA-2A/ZnT8A/IAA**	183	37.7
**Total multiple autoantibody positive (≥2)**	436	89.7
**Total prevalence of each autoantibody marker**		
**GADA**	397	81.7
**IA-2A**	371	76.3
**ZnT8A**	332	68.3
**IAA**	348	71.6
**Total identified**	474	97.5
**No detectable islet autoantibodies***	12	2.5

GADA – Glutamate decarboxylase 65 autoantibodies; IA-2A – Islet cell antigen autoantibodies; ZnT8A – Zinc transporter 8 [arginine (R)/tryptophan (W) major variants of ZnT8A] autoantibodies; IAA – Insulin autoantibodies. * Children negative for all islet autoantibodies were also negative for autoantibodies to tetraspanin 7 (TSPAN7A) and half were confirmed ICA negative.
